# The Demographic Features, Clinicopathological Characteristics and Cancer-specific Outcomes for Patients with Microinvasive Breast Cancer: A SEER Database Analysis

**DOI:** 10.1038/srep42045

**Published:** 2017-02-06

**Authors:** Wenna Wang, Wenjie Zhu, Feng Du, Yang Luo, Binghe Xu

**Affiliations:** 1 Department of Medical Oncology, National Cancer Center/Cancer Hospital, Chinese Academy of Medical Sciences and Peking Union Medical College, Beijing, 100021, China

## Abstract

To investigate the clinicopathological characteristics and survival outcomes of microinvasive breast cancer, we conducted an observational study of female diagnosed with DCIS or DCIS with microinvasion (DCISM) from 1990 to 2012 using the Surveillance, Epidemiology, and End Results (SEER) database. There were 87695 DCIS and 8863 DCISM identified. In DCISM group, patients appeared to be younger and more black patients were identified in comparison with DCIS group. Furthermore, DCISM was associated with more aggressive tumor characteristics like higher rates of oestrogen receptor (ER) and progesterone receptor (PR) negativity, HER2 positivity, and lymph node metastasis. With a median follow-up of 91 months, patients with DCISM had worse cancer-specific survival (CSS) (hazard ratio [HR], 2.475; *P* < 0.001) and overall survival (OS) (HR, 1.263; *P* < 0.001). In the multivariable analysis, microinvasion was an independent prognostic factor for worse CSS (HR, 1.919; *P* < 0.001) and OS (HR, 1.184; *P* < 0.001). The 10-year cancer-specific mortality rate was 1.49% in DCIS and 4.08% in DCISM (HR, 2.771; *P* < 0.001). The 20-year cancer-specific mortality rate was 4.00% in DCIS and 9.65% in DCISM (HR, 2.482; *P* < 0.001). Deepening understanding of the nature of microinvasive breast cancer will be valuable for clinical treatment recommendations.

Ductal carcinoma *in situ* (DCIS) of the breast is a preinvasive neoplasm originating from the abnormal proliferation of the epithelial cells without invasion beyond the basal membrane of the breast ductal system^1^. With the wide use of mammographic screening programs, the incidence of DCIS has markedly increased by five folds over the last 3 decades, accounting for approximately 20% to 25% of newly diagnosed malignancies of the breast in the United States currently^2^,^3^,^4^. It is now evident that DCIS as a precursor lesion is a heterogeneous group of lesions with diverse malignant potential^5^.

Ductal carcinoma *in situ* with microinvasion (DCISM) is an uncommon pathologic entity accounting for approximately 1% of all breast cancer cases^6^. The relative rarity and inconsistent definitions for microinvasion have contributed to the confusion regarding this entity. The American Joint Committee on Cancer Staging Manual lists “T1mic” in the TNM classification and defines microinvasion as the extension of cancer cells beyond the basement membrane into the adjacent tissue with no focus more than 1 mm in greatest dimension^7^,^8^. Although several recent studies reported on the histopathologic findings and clinical outcomes of DCISM^9^,^10^,^11^,^12^,^13^, it remains controversial whether the biologic behavior and survival outcomes of this special breast cancer subtype are distinct from those of DCIS.

Further evaluation on the impact of microinvasion on survival is essential to defining the treatment recommendations and prognosis. Therefore, a population-based study was designed to assess the differences in clinicopathologic characteristics and long-term outcomes between DCIS and DCISM using the National Cancer Institute’s Surveillance, Epidemiology, and End Results (SEER) database.

## Results

### Patient Characteristics

87695 patients with DCIS and 8863 patients with DCISM met the inclusion criteria. Demographic and clinicopathologic characteristics of the study population are summarized in Table 1. Based on the available information, significant difference of age at diagnosis, race, grade, ER status, PR status, HER2 status, lymph node status, and surgical treatment were observed between patients with microinvasive carcinoma and DCIS.

In DCISM group, more patients appeared to be younger than 40 years old (6.6% vs. 4.8%; *P* < 0.001) and more black patients(12.8% vs. 11.6%; *P* < 0.001) were identified in comparison with DCIS group. In addition, DCISM was associated with more aggressive tumor characteristics like ER negative (33.1% vs. 17.5%; *P* < 0.001), PR negative (44.9% vs. 27.3%; *P* < 0.001), HER2 positive (36.5% vs. 32.4%; *P* = 0.009) and lymph node metastasis (7.6% vs. 0%; *P* < 0.001). In terms of the treatment, 99.3% of DCISM patients underwent surgery, higher than that (97.6%) of DCIS patients (*P* < 0.001). A similar trend of radiotherapy was observed between two cohorts that 45.4% of DCISM patients and 45.7% of DCIS patients received radiation (*P* = 0.631).

### Comparison of Survival Outcomes between DCIS Patients and DCISM Patients

With a median follow-up of 91 months from diagnosis, 5922 deaths were reported in the DCIS group (n = 87695), among which 1230 deaths were related to breast cancer. 699 deaths were observed in the DCISM group (n = 8863), among which 284 deaths were attributable to breast cancer.

**Figure 1:**
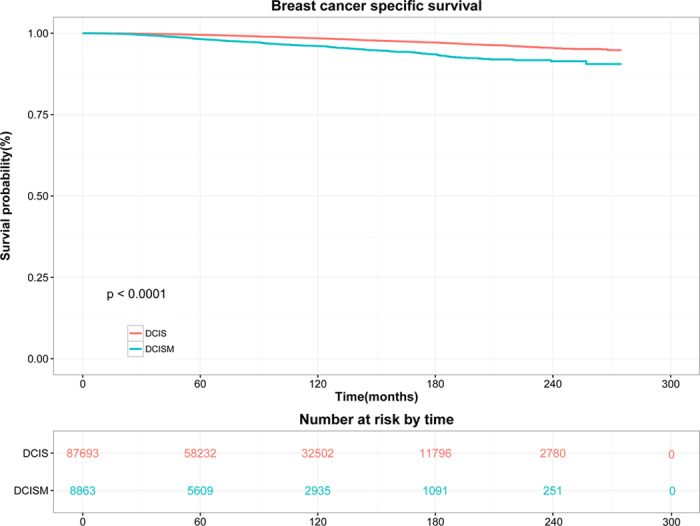
Kaplan-Meier curves and Log-rank test for breast cancer-specific survival (*P* < 0.001).

**Figure 2:**
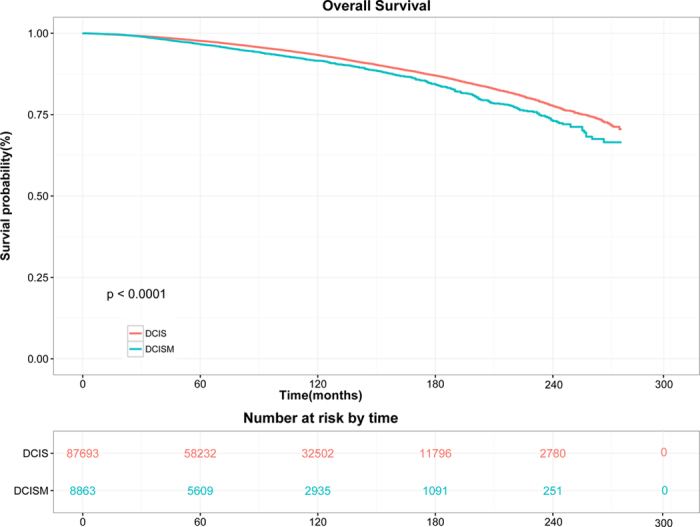
Kaplan-Meier curves and Log-rank test for overall survival (*P* < 0.001).

Survival distributions of two groups were demonstrated in Figs 1 and 2. Since a relatively small portion of overall mortality is related to breast cancer, we analyzed competing causes of death. In univariate analysis, DCISM patients was correlated with worse CSS (hazard ratio [HR], 2.475; 95% confidence interval [CI], 2.175–2.817; *P* < 0.001; log-rank *P* < 0.001; Fig. 1) and OS (HR, 1.263; 95% CI, 1.168–1.366; log-rank *P* < 0.001; Fig. 2) than the DCIS population. The 10-year CSS rate was 1.49% in DCIS and 4.08% in DCISM (HR, 2.771; 95% CI, 2.385–3.221; *P* < 0.001). The 20-year CSS rates were 4.00% and 9.65%, respectively (HR, 2.482; 95% CI, 2.180–2.825; *P* < 0.001).

Univariate and multivariate Cox proportional hazard regression models were used to calculate hazard ratio and 95% confidence interval and investigate prognostic factors that were associated with CSS and OS. As shown in Table 2, in the univariate model, it was found that microinvasive carcinoma, age at diagnosis, race, tumour grade, ER status, PR status, lymph node status, surgery treatment and radiation were significantly associated with CSS. All of these variables were included in the multivariate analysis, and microinvasion (DCISM vs. DCIS) was an independent prognostic factor for worse CSS (HR, 1.919; 95% CI, 1.643–2.240; *P* < 0.001) after adjusting for other prognostic factors. Furthermore, younger age (*P* < 0.001), black race (*P* < 0.001), higher tumour grade (*P* ≤ 0.028), lymph node metastasis (*P* < 0.001), no surgery treatment (*P* < 0.001) and no radiation (*P* < 0.001) were also independent variables to predict worse CSS. As shown in Table 3, in the univariate analysis, prognostic indicators were found to be significantly associated with OS. These factors included microinvasive carcinoma, age at diagnosis, race, ER status, PR status, lymph node status, surgery treatment and radiation. In the multivariate analysis, microinvasion (DCISM vs. DCIS) was also an independent prognostic factor for worse OS (HR, 1.184; 95% CI, 1.085–1.291; *P* < 0.001) with adjusting for other prognostic factors. In addition, older age (*P* < 0.001), black race (*P* < 0.001), lymph node metastasis (*P* ≤ 0.01), no surgery treatment (*P* < 0.001) and no radiation (*P* < 0.001) were also identified as independent prognostic variables for worse OS.

These findings were confirmed even after the weighted Cox proportional hazards regression models with the inverse probability of treatment weighting (IPTW) adjustment. IPTW-adjusted analysis for OS and IPTW-adjusted competing risk analysis for CSS suggested the mortality increased in DCISM (OS, HR, 1.138; 95% CI, 1.043–1.241; *P* < 0.001; CSS, HR, 3.801; 95% CI, 3.245–4.451; *P* < 0.001).

### Univariate and Multivariate Analyses of Prognostic Variables in DCISM Patients

To further determine the independent prognostic factors for CSS and OS in DCISM patients, both univariate and multivariate analysis were conducted (Supplementary Tables S1 and S2, available at Scientific Reports online). As shown in Supplementary Table S1, in multivariate regression model, younger age (HR, 0.544; 95% CI, 0.393–0.753; *P* < 0.001), black race (HR, 1.658; 95% CI, 1.208–2.277; *P* = 0.002), lymph node metastasis (N1: HR, 2.716, 95% CI, 1.975–3.734, *P* < 0.001; N2: HR, 5.487, 95% CI, 3.017–9.978, *P* < 0.001; and N3: HR, 20.096, 95% CI, 11.043–36.572, *P* < 0.001), no surgery treatment (HR, 6.395; 95% CI, 3.251–12.582; *P* < 0.001) and no radiation (HR, 1.390; 95% CI, 1.074–1.799; *P* < 0.001) were independent prognostic factors for worse CSS. As shown in the Supplementary Table S2, in multivariate regression model, black race (HR, 1.898; 95% CI, 1.556–2.316; *P* < 0.001), lymph node metastasis(N1: HR, 1.315, 95% CI, 1.009–1.714, *P* = 0.046; N2: HR, 2.392, 95% CI, 2.104–5.469, *P* < 0.001; and N3: HR, 7.971, 95% CI, 4.594–13.828, *P* < 0.001), no surgery treatment (HR, 3.580; 95% CI, 1.962–6.531; *P* < 0.001) and no radiation (HR, 1.377; 95% CI, 1.172–1.619; *P* < 0.001) were independent prognostic factors for worse OS. For the set of DCISM data, there were small or no number of events in the group of certain variables like HER2 variable (SEER database provided HER2 status after 2010), resulting in calculating extremely small or large HRs in the univariate and multivariate analysis.

### Subgroup Analysis

**Figure 3:**
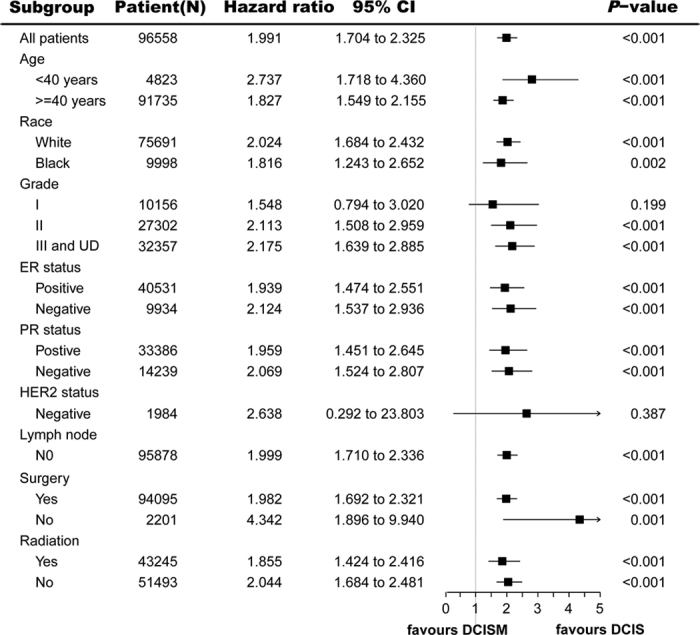
Forest plot of hazard ratios and 95% CIs for breast cancer-specific survival in subgroups. HR = hazard ratio, CI = confidence interval, DCIS = ductal carcinoma *in situ*, DCISM = ductal carcinoma *in situ* with microinvasion, ER = oestrogen receptor, HER2 = human epidermal growth factor receptor 2, PR = progesterone receptor, UD = undifferentiated.

**Figure 4:**
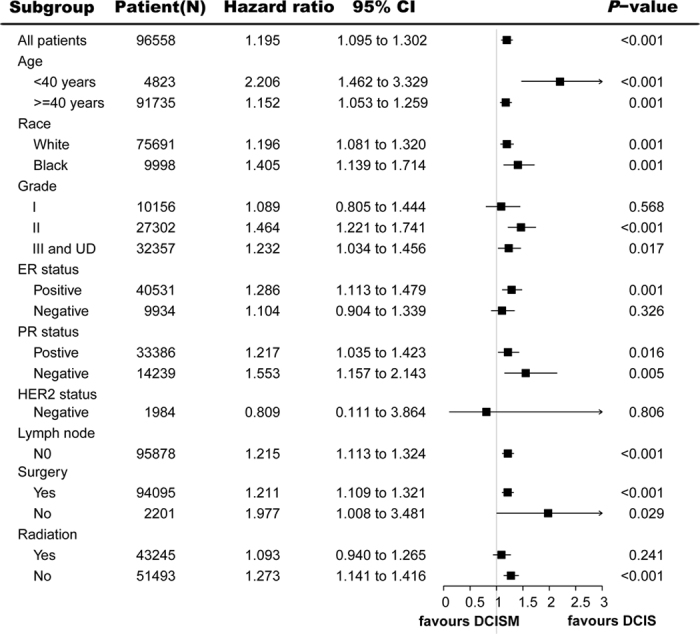
Forest plot of hazard ratios and 95% CIs for overall survival in subgroups. HR = hazard ratio, CI = confidence interval, DCIS = ductal carcinoma *in situ*, DCISM = ductal carcinoma *in situ* with microinvasion, ER = oestrogen receptor, HER2 = human epidermal growth factor receptor 2, PR = progesterone receptor, UD = undifferentiated.

Exploratory subgroup analyses were carried out for the following factors: age, race, grade, ER status, PR status, HER2 status, lymph node status, surgery and radiotherapy. Forest plots show hazard ratios and 95% CIs for CSS (Fig. 3) and OS (Fig. 4) in the subgroups. Subgroup analyses of CSS and OS were consistent with the overall estimate in most patient subgroups. Most of the estimated HRs were >1.0, thus favouring DCIS over DCISM, and the 95% CIs lower limits were more than 1.0. The similarity of the estimated HRs across the subgroups supports the robustness of the primary analysis. Cox regression showed in Fig. 3 there were significant differences between HRs of DCISM versus DCIS for CSS in subgroups of age (<40 years or ≥40 years), race (white or black), gradeII, grade III and UD, ER (positive or negative), PR (positive or negative), no lymph node, surgery (yes or no) and radiotherapy (yes or no). Moreover, as shown in Fig. 4, HRs for OS in subgroups of age (<40 years or ≥40 years), race (white or black), gradeII, grade III and UD, ER positive, PR negative, no lymph node, surgery (yes or no) and no radiotherapy were significantly different between DCISM and DCIS. Some results must be interpreted with caution because of the small numbers of events in some subgroups like HER2 negative.

## Discussion

Over the past three decades, more early stage breast carcinoma including ductal carcinoma *in situ* and microinvasive carcinoma have been detected and diagnosed with the wide use of mammographic screening programs and the advances in mammographic techniques. Microinvasive carcinoma is rare and there are controversial results reported on the survival outcomes of this special breast cancer subtype compared with those of DCIS^12^,^13^,^14^,^15^,^16^,^17^. Deepening the understanding of the nature of microinvasive breast cancer and identifying the long-term outcomes of DCISM would be valuable for better clinical treatment recommendations. This study using SEER database is to date the first and largest study to compare the clinicopathology and long-term prognosis between DCIS and DCISM in a U.S. population with a median follow-up of 91 months. Our analysis shows that DCISM patients have worse cancer-specific survival and overall survival in the univariate analysis (CSS: HR, 2.475; 95%CI, 2.175–2.817; *P* < 0.001; OS: HR, 1.263; 95% CI, 1.168–1.366; *P* < 0.001), and in the multivariate analysis, microinvasion is an independent prognostic factor for worse CSS (HR, 1.919; 95% CI, 1.643–2.240; *P* < 0.001) and OS (HR, 1.184; 95% CI, 1.085–1.291; *P* < 0.001). Based on the available information, in DCISM group, patients appear to be younger and more black patients are identified in comparison with DCIS group. Furthermore, DCISM is characterized by more aggressive clinicopathologic features. Further multivariate analyses show lymph node metastasis, no surgery treatment and no radiation are independent prognostic factor for worse CSS and OS in DCISM patients.

Our current analysis showed the 10-year and 20-year breast cancer-specific mortality after a diagnosis of DCIS were 1.49% and 4.00%, respectively, consistent with the results reported in previous studies^18^,^19^,^20^. One observational study in women diagnosed of DCIS from 1988 to 2011 in the SEER18 registries database reported that the 10-year breast cancer-specific mortality rate was 1.1% and 20-year mortality rate was 3.3% with a mean follow-up of 7.5 years^18^. In another previous study based on data from the SEER database, the 10-year breast cancer mortality rate was 3.4% for women who diagnosed of DCIS from 1978 to 1983 and 1.9% for women who had a diagnosis from 1984 to 1989^20^. Previous studies on the prognosis of DCISM had limited sample size (fewer than 300 cases) with variable degrees of histologic sampling. Consequently, the clinical significance of microinvasion remains unclear. This study with 8863 DCISM cases suggested DCISM had worse cancer-specific survival and overall survival in comparison with DCIS. The breast cancer-specific mortality rate for DCISM was 4.08% at 10 years and was 9.65% at 20 years, higher than those for DCIS. Several studies suggested that the biologic behavior and survival outcomes of DCISM were intermediate between those of DCIS and invasive breast cancer^13^,^14^. De Mascarel *et al*. evaluated the clinical significance of microinvasion in the ever largest series of patients with DCISM (243 cases)^13^. In the study, patients were divided in two distinct pathologic groups: type 1 with isolated cells, and type 2 with clusters of cells. Overall survival rates were not significantly different in DCIS and DCISM type 1 patients at 10 years (96.5% and 96.3%, respectively). Whereas it was significantly different between DCIS and DCIS-MI type 2 patients (96.5% vs. 88.4%; *P* < 10^4^). However, there has also been disagreement on the impact of microinvasion on survival^12^,^15^,^16^,^17^. In a study enrolling 72 patients with DCISM and 321 patients with DCIS, there was on significant differences in outcomes for the two cohorts^12^. The results showed, the 10-year OS rate for DCISM and DCIS patients was 93.2% and 95.7% (*P* = 0.95), respectively, with a median follow-up of 8.94 years. Wang *et al*. also reported that the outcomes of patients with microinvasive carcinoma were similar to those with DCIS. The 5-year OS rate for microinvasive carcinoma and DCIS patients was 99.0 and 99.2%, respectively^17^. However, these studies enrolled few patients with DCISM. Considering the low number of patients, these studies may be underpowered to detect a significant difference in the long term outcomes with DCISM. Moreover, the reliability of these conclusions may also be limited by the relatively short follow-up duration.

On the basis of our large dataset, 7.6% of DCISM had lymph node metastases. In our multivariate analysis, lymph node metastasis was an independent prognostic factor for worse CSS (*P* < 0.001) and OS (*P* ≤ 0.010). The incidence of pathologically positive axillary lymph node metastases for patients with microinvasive ductal carcinoma of the breast has been reported as 0–20%^21^,^22^,^23^,^24^,^25^,^26^,^27^,^28^,^29^,^30^,^31^,^32^,^33^,^34^,^35^,^36^,^37^. Maibenco *et al*. reported the frequency of nodal metastases was 10.5% in the 1229 female patients with microinvasive breast cancer who underwent axillary staging in the SEER database from 1997 through 2003^21^. Five-year survival rate was 99% among lymph node negative cases and 95% among lymph node positive cases. In univariate analysis, survival varied with the lymph node status (*P* = 0.004). To some extent, the presence of lymph node metastases results in the poor prognosis of microinvasive breast cancer. These patients should receive more aggressive treatment in the clinical practice.

Previous studies have suggested that DCISM may represent a distinct entity with more aggressive pathological features associated with worse survival outcomes^11^,^38^. It has been proven that hormonal receptors negativity and HER2 overexpression promoted breast cancer invasion and metastasis^39^,^40^,^41^,^42^,^43^. Our analysis showed, compared with DCIS, DCISM was more ER negative (33.1% vs. 17.5%; *P* < 0.001), PR negative (44.9% vs. 27.3%; *P* < 0.001), and HER2 positive (36.5% vs. 32.4%; *P* = 0.009). Margalit *et al*. reported 39% ER negative and 49% HER2 positive in 83 consecutive patients with microinvasive breast cancer from 1997 to 2005, more frequent than in DCIS^11^. Another study reported that the incidence of ER negative-HER2 positive type in DCISM was 46.9%, significantly higher than in DCIS (46.9% vs. 8.7%; *P* < 0.001)^44^. In a retrospective study with 271 DCIS and 67 DCISM, less luminal-like tumors were observed in DCISM, whereas more HER2 positive and basal-like tumors were identified in DCISM compared with DCIS (*P* = 0.039)^38^. Based on the results of the above studies, we could hypothesize that hormonal receptors negativity and HER2 overexpression might play an important role in the development of microinvasion in DCIS. On the contrary, DCIS with positive hormonal receptors and negative HER2 expression may stay stable for a long duration because of the weakness of the initial invasion. Furthermore, the difference in pathologic characteristics between DCIS and DCISM justifies the different strategies in management.

In order to precisely estimate the mortality, it is necessary to conduct a large cohort study with an extended period since death events rarely happen in DCIS or DCISM. Despite the utility of large, high quality cancer data registries such as the SEER database, there are several limitations in our study, including lack of certain characteristics such as surgical margin status, adjuvant endocrine therapy, the process of adjuvant chemotherapy and body mass index. Additionally, key data such as tumor grade and hormone receptor status were unavailable for approximately 30–50% of DCIS or DCISM, and HER2 status were missing in more than 90% patients. Another important limitation is that the SEER registry records multiple primary cancers but not recurrences which may result in inaccurate estimation on disease free survival.

In conclusion, the clinicopathological characteristics of breast cancer patients with microinvasion are more aggressive than those of DCIS. Furthermore, microinvasion is an independent prognostic factor for worse CSS and OS. The direction in the future will be to further explore and differentiate those subtypes of microinvasive carcinoma associated with a higher incidence of recurrence or progression to invasive disease in order to tailor treatment strategy accordingly.

## Methods

### Database

Data for this study were obtained from the recent SEER 18 registries research database (November 2014 Submission). The SEER18 database contains data from the SEER13 registries (Atlanta, Connecticut, Detroit, Hawaii, Iowa, New Mexico, San Francisco-Oakland, Seattle-Puget Sound, Utah, Los Angeles, San Jose-Monterey, rural Georgia, and the Alaska Native Tumor Registry) and the registries of greater California, Kentucky, Louisiana, New Jersey, and greater Georgia. SEER database of the National Cancer Institute (NCI) is the largest population-based cancer registry in the United States, which covers approximately 28% of the population (http://seer.cancer.gov/about/).

### Study Population

We conducted a retrospective cohort study and SEER*Stat (version 8.2.1) was used to generate a case listing. To identify the eligible DCIS cohort, the inclusion criteria included females aged 20 to 69 years old; the first and only cancer diagnosis with stage Tis breast cancer between 1990 and 2012; patients with the International Classification of Diseases for Oncology Version 3 (ICD-O-3) codes of 8201/2 (Cribriform carcinoma *in situ*), 8230/2 (Duct carcinoma *in situ*, solid type), 8500/2 (Intraductal carcinoma, non-infiltrating), 8501/2 (Comedocarcinoma, non-infiltrating), 8503/2 (Noninfiltrating intraductal papillary adenocarcinoma), 8201/2 (Cribriform carcinoma *in situ*) and 8507/2 (Intraductal micropapillary carcinoma); and without ductal carcinoma with microinvasion. To identify the eligible DCISM cohort, the inclusion criteria included females aged 20 to 69 years old; the first and only cancer diagnosis with stage T1mic breast cancer between 1990 and 2012; patients with ICD-O-3 codes of 8201/3 (Cribriform carcinoma), 8230/3 (Solid carcinoma), 8500/3 (Infiltrating duct carcinoma), 8501/3 (Comedocarcinoma), 8503/3 (Intraductal papillary adenocarcinoma with invasion), and 8507/3 (Ductal carcinoma, micropapillary). Patients for whom DCIS or DCISM was not the first and only cancer diagnosis were excluded from analysis. Patients without microscopic confirmation of the diagnosis and those identified at autopsy or on death certificate only were also excluded from the analyses. Together, 87695 patients with DCIS and 8863 patients with DCISM were eligible for the study.

We signed Data-Use Agreement for the SEER 1973–2012 Research Data File and obtained permission to access the SEER cancer registries. Since the present study is a database-based analysis rather than experimental research on humans, informed patient consent is not needed. Our study was approved by independent ethics committees of Cancer Institute and Hospital, Chinese Academy of Medical Science. The methods were carried out in accordance with the relevant guidelines and regulations.

### SEER Variables and Covariates

Demographic variables included age, race (white, black, others, or unknown). Tumor-specific variables included: grade (I, II, III, undifferentiated, or unknown), estrogen receptor (ER) status (positive, negative, borderline, or unknown), progesterone receptor (PR) status (positive, negative, borderline, or unknown), epidermal growth factor receptor 2 (HER2) status (positive, negative, borderline, or unknown), and lymph node metastasis (N0, N1, N2, N3, or Nx). Treatment-related information included: surgery (performed, not performed, or unknown) and radiation (performed, not performed, or unknown). Survival- specific variables included survival months, vital status recode (alive or dead), cause-specific death, and other cause of death.

### Statistical analysis

The distribution of patient and clinicopathologic characteristics between two groups was compared using Pearson’s Chi-square test or Fisher exact test, as appropriate. Cancer-specific survival (CSS) was defined as the time from diagnosis to death from breast cancer. Overall survival (OS) was calculated from the date of diagnosis to the date of death from any cause. Kaplan-Meier estimates and Cox regression analyses of CSS and OS were done. The log-rank test was used to compare the distribution between the DCIS and DCISM groups. Fine and Gray’s competing-risks regression models were conducted to assess cancer-specific survival (CSS). Univariate and multivariate Cox proportional hazard regression models were used to calculate hazard ratio (HR) and 95% confidence interval (CI) and identify factors that are associated with CSS and OS.

In order to adjust potential confounders by indication (covariates: age, race, grade, ER status, PR status, HER2 status, lymph node status, surgery and radiotherapy), the weighted Cox proportional hazards regression models with the inverse probability of treatment weighting (IPTW) were conducted. Subgroup analyses were undertaken to investigate the effect of multiple prognostic factors on CSS and OS by use of a Cox regression model. For the subgroup analysis of survival, the HR and 95% CI within each subgroup were summarized and displayed in the forest plot.

All statistical analyses were performed using R statistical package version 3.1.1 (R Project for Statistical Computing, Vienna, Austria) or SAS version 9.2 (SAS Institute Inc., Cary, North Carolina). All analyses were 2-sided and statistical significance was defined as *P* < 0.05.

## Additional Information

**How to cite this article**: Wang, W. *et al*. The Demographic Features, Clinicopathological Characteristics and Cancer-specific Outcomes for Patients with Microinvasive Breast Cancer: A SEER Database Analysis. *Sci. Rep.* **7**, 42045; doi: 10.1038/srep42045 (2017).

**Publisher's note:** Springer Nature remains neutral with regard to jurisdictional claims in published maps and institutional affiliations.

## Supplementary Material

Supplementary Tables

## Figures and Tables

**Table 1:** Patient characteristics of the study population.

	DCIS N = 87695	(%)	DCISM N = 8863	(%)	*P* Value
Age at diagnosis, years					<0.001
20–29	249	0.3	43	0.5	
30–39	3987	4.5	544	6.1	
40–49	25115	28.6	2418	27.3	
50–59	31665	36.1	3223	36.4	
60–69	26679	30.4	2635	29.7	
Race					<0.001
white	68939	88.6	6752	87.2	
black	9004	11.6	994	12.8	
other or unknown	9752		1117		
Histologic subtype					<0.001
cribriform	9375	10.7	58	0.7	
solid type	6098	7.0	44	0.5	
ductal carcinoma, NOS	51120	58.3	7949	89.7	
comedonecrosis	15209	17.3	691	7.8	
papillary	3154	3.6	108	1.2	
micropapillary	2739	3.1	13	0.2	
Lymph node					<0.001
N0	87692	100	8186	92.4	
N1	0	0	569	6.4	
N2	0	0	76	0.9	
N3	0	0	32	0.4	
unknown	3		0		
Grade					<0.001
I	9140	14.3	1016	17.7	
II	25129	39.3	2173	37.8	
III and UD	29799	46.5	2558	44.5	
unknown	23627		3116		
ER status					
positive	35986	82.2	4545	66.3	
negative	7665	17.5	2269	33.1	
Borderline	124	0.3	41	0.6	
unknown	43920		2008		
PR status					<0.001
positive	29783	72.2	3603	54.2	
negative	11252	27.3	2987	44.9	
Borderline	223	0.5	57	0.9	
unknown	46437		2216		
HER2 status					0.009
positive	708	32.4	416	36.5	
negative	1320	60.3	664	58.3	
Borderline	160	7.3	59	5.2	
unknown	85507		7724		
radiation					0.631
yes	39300	45.7	3945	45.4	
no	46749	54.3	4744	54.6	
unknown	1646		174		
surgery					<0.001
yes	85306	97.6	8789	99.3	
no	2137	2.4	64	0.7	
unknown	252		10		
Vital status					<0.001
alive	81773	93.2	8164	92.1	
dead of other cause	4692	5.4	415	4.7	
breast cancer-specific dead	1230	1.4	284	3.2	

DCIS = ductal carcinoma *in situ*, DCISM = ductal carcinoma *in situ* with microinvasion, NOS = not otherwise specified, ER = estrogen receptor, PR = progesterone receptor, HER2 = epidermal growth factor receptor 2, UD = undifferentiated.

**Table 2:** Univariate and multivariate analysis of breast cancer-specific survival.

Variables	Univariate analysis			Multivariate analysis		
	HR	(95% CI)	*P*-value	HR	(95% CI)	*P*-value
Histology type						
DCIS	ref					
DCISM	2.475	(2.175–2.817)	<0.001	1.919	(1.643–2.240)	<0.001
Age						
<40	ref					
≥40	0.627	(0.527–0.745)	<0.001	0.726	(0.609–0.864)	<0.001
Race						
white	ref					
black	2.240	(1.962–2.558)	<0.001	2.131	(1.865–2.434)	<0.001
other	0.978	(0.817–1.171)	0.807	0.978	(0.817–1.171)	0.811
unknown	0.785	(0.373–1.651)	0.523	0.557	(0.264–1.176)	0.125
Grade						
I	ref					
II	1.324	(1.042–1.684)	0.022	1.309	(1.029–1.665)	0.028
III and UD	1.754	(1.393–2.209)	<0.001	1.702	(1.349–2.148)	<0.001
unknown	1.617	(1.289–2.027)	<0.001	1.560	(1.243–1.957)	<0.001
ER status						
positive	ref					
negative	1.650	(1.380–1.973)	<0.001	1.189	(0.933–1.511)	0.161
Borderline	2.107	(0.940–4.725)	0.070	1.127	(0.468–2.713)	0.790
unknown	0.900	(0.793–1.021)	0.102	0.998	(0.675–1.477)	0.994
PR status						
positive	ref					
negative	1.584	(1.328–1.890)	<0.001	1.190	(0.938–1.511)	0.151
Borderline	2.493	(1.326–4.686)	0.005	2.138	(1.076, 4.246)	0.030
unknown	0.925	(0.807–1/060)	0.261	1.038	(0.696, 1.549)	0.855
HER2						
positive	ref					
negative	0.852	(0.142–5.101)	0.861	1.148	(0.191, 6.884)	0.880
Borderline	2.296	(0.208–25.314)	0.497	3.429	(0.310–37.910)	0.315
unknown	0.501	(0.124–2.021)	0.331	0.859	(0.212–3.476)	0.831
Lymph node						
N0	ref					
N1	6.181	(4.675–8.172)	<0.001	2.828	(2.077–3.850)	<0.001
N2	10.521	(5.961–18.571)	<0.001	5.743	(3.194–10.324)	<0.001
N3	36.481	(21.128–62, 990)	<0.001	19.573	(11.069–34.612)	<0.001
surgery						
yes	ref					
no	3.635	(2.956–4.470)	<0.001	3.887	(3.149–4.798)	<0.001
unknown	3.630	(1.811–7.275)	<0.001	4.027	(1.998–8.119)	<0.001
radiation						
yes	ref					
no	1.344	(1.207–1.496)	<0.001	1.289	(1.156–1.437)	<0.001
unknown	1.151	(0.758–1.748)	0.510	0.892	(0.583–1.365)	0.598

HR = hazard ratio, CI = confidence interval, DCIS = ductal carcinoma *in situ*, DCISM = ductal carcinoma *in situ* with microinvasion, ER = oestrogen receptor, HER2 = human epidermal growth factor receptor 2, PR = progesterone receptor, UD = undifferentiated. Multivariate analysis included histology, age, race, grade, ER status, PR status, HER2 status, lymph node status, surgery and radiation.

**Table 3:** Univariate and Multivariate Analysis of overall survival.

Variables	Univariate analysis			Multivariate analysis		
	HR	(95% CI)	P-value	HR	(95% CI)	P-value
Histology type						
DCIS	ref					
DCISM	1.263	(1.168–1.366)	<0.001	1.184	(1.085–1.291)	<0.001
Age						
<40	ref					
≥40	2.097	(1.825–2.410)	<0.001	2.229	(1.939–2.563)	<0.001
Race						
white	ref					
black	1.881	(1.760–2.011)	<0.001	1.885	(1.764–2.015)	<0.001
other	0.750	(0.683–0.825)	<0.001	0.760	(0.692–0.836)	<0.001
unknown	0.521	(0.340–0.800)	0.003	0.463	(0.294–0.696)	<0.001
Grade						
I	ref					
II	1.000	(0.904–1.105)	0.995	1.025	(0.927–1.133)	0.629
III and UD	1.000	(0.906–1.104)	0.996	1.022	(0.925–1.129)	0.669
unknown	1.121	(1.021–1.231)	0.017	1.123	(1.022–1.233)	0.016
ER status						
positive	ref					
negative	1.198	(1.086–1.321)	<0.001	1.502	(0.924–1.198)	0.446
Borderline	1.305	(0.797–2.136)	0.290	1.013	(0.601–1.708)	0.962
unknown	1.021	(0.960–1.085)	0.518	1.147	(0.939–1.401)	0.178
PR status						
positive	ref					
negative	1.226	(1.117–1.346)	<0.001	1.165	(1.030–1.319)	0.015
Borderline	1.302	(0.854–1.986)	0.220	1.231	(0.788–1.923)	0.361
unknown	1.033	(0.967–1.104)	0.331	0.912	(0.743–1.119)	0.375
HER2						
positive	ref					
negative	1.024	(0.343–3.054)	0.967	1.119	(0.375–3.343)	0.840
Borderline	0.933	(0.109–7.950)	0.949	1.006	(0.117–8.611)	0.996
unknown	1.025	(0.425–2.470)	0.956	1.188	(0.492–2.866)	0.702
Lymph node						
N0	ref					
N1	1.695	(1.322–2.172)	<0.001	1.409	(1.086–1.828)	0.010
N2	3.471	(2.186–5.513)	<0.001	3.437	(2.146–5.506)	<0.001
N3	8.951	(5.298–15.123)	<0.001	8.221	(4.831–13.989)	<0.001
surgery						
yes	ref					
no	1.881	(1.645–2.151)	<0.001	1.837	(1.605–2.103)	<0.001
unknown	2.075	(1.338–3.220)	0.001	1.997	(1.284–3.106)	0.002
radiation						
yes	ref					
no	1.238	(1.176–1.302)	<0.001	1.228	(1.167–1.293)	<0.001
unknown	1.223	(1.008–1.483)	0.041	1.156	(0.951–1.404)	0.145

HR = hazard ratio, CI = confidence interval, DCIS = ductal carcinoma *in situ*, DCISM = ductal carcinoma *in situ* with microinvasion, ER = oestrogen receptor, HER2 = human epidermal growth factor receptor 2, PR = progesterone receptor, UD = undifferentiated. Multivariate analysis included histology, age, race, grade, ER status, PR status, HER2 status, lymph node status, surgery and radiation.
