# Supplementary material for: The Demographic Features, Clinicopathological Characteristics and Cancer-specific Outcomes for Patients with Microinvasive Breast Cancer: A SEER Database Analysis
Source: Sci Rep. 2017 Feb 6;7:42045. doi: 10.1038/srep42045 (PMC5292735; doi:10.1038/srep42045)
Supplement: Supplementary Tables [file srep42045-s1.pdf]

The Demographic Features, Clinicopathological Characteristics and Cancer-specific Outcomes for Patients with Microinvasive Breast Cancer: A SEER Database Analysis

Wenna Wang, MD[1], Wenjie Zhu, MD[1], Feng Du, MD[1], Yang Luo, MD[1], Binghe Xu, MD[1*]

[1]Department of Medical Oncology, National Cancer Center/Cancer Hospital, Chinese Academy of Medical Sciences and Peking Union Medical College, Beijing, 100021, China.

***Corresponding author**: Binghe Xu, Department of Medical Oncology, National Cancer Center/Cancer Hospital, Chinese Academy of Medical Sciences and Peking Union Medical College, No.17 Panjiayuan Nanli, Chaoyang District, Beijing, 100021, China. Telephone: 8610-87788826；Fax: 8610-87715711；E-mail: xubinghe@medmail.com.cn

Supplementary Table S1. Univariate and multivariate analysis for cancer-specific survival in DCISM patients

Variables	Univariate analysis			Multivariate analysis		
	HR	(95% CI)	*P*-value	HR	(95% CI)	*P*-value
Age						
<40	ref					
≥40	0.404	(0.294-0.554)	<0.001	0.544	(0.393-0.753)	<0.001
Race						
white	ref					
black	1.929	(1.416-2.629)	<0.001	1.658	(1.208-2.277)	0.002
other	0.922	(0.622-1.369)	0.689	0.926	(0.623-1.376)	0.704
unknown	<0.001	(0-999)	0.944	<0.001	(0-999)	0.946
Grade						
Ⅰ	ref					
Ⅱ	2.005	(1.164-3.454)	0.012	1.564	(0.903-2.711)	0.111
Ⅲ and UD	2.152	(1.268-3.654)	0.005	1.556	(0.904-2.681)	0.111
unknown	1.666	(0.983-2.824)	0.058	1.417	(0.830-2.418)	0.201
ER status						
positive	ref					
negative	1.458	(1.102-1.929)	0.008	1.151	(0.784-1.690)	0.472
Borderline	1.864	(0.591-5.877)	0.288	1.309	(0.380-4.514)	0.670
unknown	0.938	(0.703-1.251)	0.662	0.985	(0.439-2.207)	0.970
PR status						
positive	ref					
negative	1.487	(1.118-1.978)	0.006	1.305	(0.885-1.924)	0.179
Borderline	2.238	(0.821-6.101)	0.115	2.257	(0.768-6.632)	0.139
unknown	0.975	(0.724-1.313)	0.868	1.152	(0.517-2.568)	0.729
HER2						
positive	ref					
negative	999	(0-999)	0.924	999	(0-999)	0.925
Borderline	1.003	(0-999)	1.000	1.319	(0-999)	0.999
unknown	999	(0-999)	0.927	999	(0-999)	0.929
Lymph node						
N0	ref					
N1	3.406	(2.507-4.626)	<0.001	2.716	(1.975-3.734)	<0.001
N2	5.791	(3.235-10.365)	<0.001	5.487	(3.017-9.978)	<0.001
N3	19.307	(11.021-33.825)	<0.001	20.096	(11.043-36.572)	<0.001
surgery						
yes	ref					
no	6.710	(3.451-13.043)	<0.001	6.395	(3.251-12.582)	<0.001
unknown	6.681	(0.936-47.678)	0.058	4.786	(0.657-34.868)	0.122
radiation						
yes	ref					
no	1.420	(1.107-1.821)	0.006	1.390	(1.074-1.799)	0.012

unknown	1.744	(0.847-3.589)	0.131	0.936	(0.438-2.001)	0.864

HR = hazard ratio, CI = confidence interval, DCIS = ductal carcinoma in situ, DCISM = ductal carcinoma in situ with microinvasion, ER = oestrogen receptor, HER2 = human epidermal growth factor receptor 2, PR = progesterone receptor, UD = undifferentiated.

Supplementary Table S2. Univariate and multivariate analysis for overall survival in DCISM patients

Variables	Univariate analysis			Multivariate analysis		
	HR	(95% CI)	*P*-value	HR	(95% CI)	*P*-value
Age						
<40	ref					
≥40	0.967	(0.732-1.276)	0.811	1.146	(0.864-1.522)	0.344
Race						
white	ref					
black	1.945	(0.598-2.368)	<0.001	1.898	(1.556-2.316)	<0.001
other	0.685	(0.514-0.913)	0.010	0.689	(0.516-0.919)	0.011
unknown	<0.001	(0-999)	0.919	<0.001	(0-999)	0.897
Grade						
Ⅰ	ref					
Ⅱ	1.309	(0.971-1.766)	0.078	1.187	(0.878-1.606)	0.265
Ⅲ and UD	1.147	(0.854-1.542)	0.361	1.046	(0.773-1.416)	0.769
unknown	1.184	(0.892-1.571)	0.242	1.118	(0.839-1.489)	0.447
ER status						
positive	ref					
negative	1.023	(0.845-1.238)	0.818	0.851	(0.661-1.096)	0.213
Borderline	0.601	(0.192-1.876)	0.380	0.422	(0.130-1.368)	0.150
unknown	0.980	(0.825-1.165)	0.821	0.841	(0.530-1.334)	0.462
PR status						
positive	ref					
negative	1.174	(0.971-1.419)	0.097	1.331	(1.037-1.709)	0.025
Borderline	1.254	(0.557-2.823)	0.584	1.645	(0.712-3.800)	0.244
unknown	1.065	(0.889-1.275)	0.497	1.277	(0.806-2.025)	0.298
HER2						
positive	ref					
negative	1.871	(0.195-17.985)	0.588	2.186	(0.227-21.087)	0.499
Borderline	0.002	(0-999)	0.909	<0.001	(0-999)	0.950
unknown	1.960	(0.272-14.115)	0.504	2.208	(0.306-15.918)	0.432
Lymph node						
N0	ref					
N1	1.457	(1.124-1.889)	0.004	1.315	(1.009-1.714)	0.046
N2	2.988	(1.870-4.777)	<0.001	2.392	(2.104-5.469)	<0.001
N3	7.667	(4.511-13.029)	<0.001	7.971	(4.594-13.828)	<0.001

surgery						
yes	ref					
no	3.616	(1.992-6.565)	<0.001	3.580	(1.962-6.531)	<0.001
unknown	3.199	(0.449-22.766)	0.246	2.362	(0.330-16.927)	0.392
radiation						
yes	ref					
no	1.337	(1.141-1.566)	<0.001	1.377	(1.172-1.619)	<0.001
unknown	1.567	(0.970-2.531)	0.066	1.194	(0.730-1.952)	0.480

HR = hazard ratio, CI = confidence interval, DCIS = ductal carcinoma in situ, DCISM = ductal carcinoma in situ with microinvasion, ER = oestrogen receptor, HER2 = human epidermal growth factor receptor 2, PR = progesterone receptor, UD = undifferentiated.
